# Vascularized lymph node transfer (VLNT) versus lymphaticovenous anastomosis (LVA) for chronic breast cancer-related lymphedema (BCRL): a retrospective cohort study of effectiveness over time

**DOI:** 10.1007/s10549-024-07567-5

**Published:** 2024-12-10

**Authors:** Elisabeth A. Kappos, Adriano Fabi, Florian S. Halbeisen, Alina Abu-Ghazaleh, Julia Stoffel, Birgit Aufmesser-Freyhardt, Julia Bukowiecki, Tristan M. Handschin, Christoph Andree, Martin D. Haug, Dirk J. Schaefer, Sonia Fertsch, Katrin Seidenstücker

**Affiliations:** 1 Department of Plastic, Reconstructive, Aesthetic and Hand Surgery, University Hospital of Basel, Spitalstrasse 21, 4031 Basel, Switzerland; 2 Faculty of Medicine, University of Basel, Basel, Switzerland; 3 Breast Center, University Hospital of Basel, Basel, Switzerland; 4 Faculty of Medicine, University of Zurich, Zurich, Switzerland; 5 Surgical Outcome Research Center, Department of Clinical Research, University Hospital Basel and University of Basel, Basel, Switzerland; 6 Department of Plastic, Reconstructive and Aesthetic Surgery, Sana Hospital Düsseldorf, Düsseldorf, Germany; 7 Faculty of Health, University Witten-Herdecke, Witten, Germany; 8 Breast Center, University Hospital Düsseldorf, Düsseldorf, Germany

**Keywords:** Breast cancer-related lymphedema, Lymphedema, Breast cancer, Lymphatic surgery, Vascularized lymph node transfer, Lymphaticovenous anastomosis

## Abstract

**Purpose:**

Microsurgical reconstruction, including vascularized lymph node transfer (VLNT) and lymphaticovenous anastomosis (LVA), have emerged as promising treatment options for chronic breast cancer-related lymphedema (BCRL). Despite their clinical relevance, the precise timelines for patient improvement following these interventions remain rather unexplored. Therefore, the goal of this study was to compare the long-term outcomes and improvement patterns over time of VLNT versus LVA to lay open potential differences and aid in personalized counseling of future patients.

**Methods:**

A prospectively maintained, encrypted database was analyzed for patients with chronic BCRL treated with either VLNT or LVA with a minimum follow-up of one year. Patient-specific variables, such as body weight and circumferential arm measurements at distinct locations on both arms were documented preoperatively and on regular postoperative outpatient follow-ups.

**Results:**

This study comprised 112 patients, of which 107 patients fully completed the one-year follow-up period. Both VLNT and LVA achieved significant arm size reductions. LVA showed an early peak in effectiveness within the first three months, followed by a subsequent decrease and eventual stabilization. Contrarily, VLNT exhibited a distinct pattern with two significant peaks at three and eighteen months.

**Conclusions:**

VLNT and LVA are both effective in long-term lymphedema management, yet they demonstrate marked differences in the timing of improvement. VLNT shows a delayed but more durable response, in contrast to the greater but shorter-lasting surge in effectiveness achieved by LVA. Interestingly, VLNT demonstrates an earlier onset of therapeutic impact than previously understood.

## Introduction

Chronic breast cancer-related lymphedema (BCRL) affects approximately 20% of breast cancer survivors, manifesting as an accumulation of protein-rich interstitial fluid within the suprafascial tissue of the arm due to compromised lymphatic drainage [1–3]. It is commonly associated with debilitating physical and psychological repercussions, including pain, numbness, infections, body dysmorphia and even depression [4–8]. Complex physical decongestion therapy (CDT) offers transient symptomatic relief but does not restore lymphatic outflow capacity, necessitating lifelong intensive management [9, 10]. In contrast, microsurgical interventions have efficiently shown promising long-term outcomes by tackling the underlying pathophysiology of chronic BCRL [10–12]. Among these interventions, lymphaticovenous anastomosis (LVA) and vascularized lymph node transfer (VLNT) have become well-established microsurgical procedures with proven effectiveness [13–20].

LVA is a minimally invasive procedure, which allows for lymphatic outflow reconstruction through one or more anastomoses between lymphatic vessels and nearby subdermal venules [13–15]. The immediately functional anastomosis is believed to achieve rapid improvements after surgery and is typically employed in milder lymphedema cases, provided that there are patent lymphatic vessels present [21, 22]. However, LVA’s patency and its associated long-term effectiveness are subjects of ongoing research, with studies suggesting occlusion of 20% to 40% of anastomoses within the first year [15, 23].

In contrast, VLNT involves the autologous microsurgical transplantation of lymph nodes from an unaffected region to the lymphedematous site with subsequent vascular anastomosis to enable reestablishment of lymphatic outflow [24–26]. Contrary to LVA, it does not require patent lymphatic vessels and has also demonstrated effectiveness in more advanced stages of lymphedema [20, 27]. Both techniques can be combined to optimize outcomes in lymphedema management [22, 28]. VLNT is thought to yield greater clinical improvements than LVA, primarily through secretion of vascular endothelial growth factor C (VEGF-C), which promotes the gradual establishment of novel lymphatic networks [29–31]. This concept is validated by indocyanine green studies, which have demonstrated active transport of interstitial lymphatic fluid through the transplanted lymph node into the pedicle vein [31, 32]. Given that lymphangiogenesis is generally recognized as a time-consuming process, the effectiveness of VLNT is hypothesized to progressively increase over time.

While a comparative analysis of their effectiveness over time would markedly enhance the accuracy of patient information, their temporal differences remain notably unexplored in the current literature. Therefore, the goal of this study was to compare temporal improvement profiles and long-term effectiveness of VLNT and LVA, hence improving counseling for patients suffering from chronic BCRL and enabling a more accurate management of expectations in their individual treatment approaches.

## Methods

### Study design

A retrospective analysis was performed on a prospectively maintained encrypted database of patients treated for chronic BCRL at a tertiary referral center. The inclusion criteria comprised of patients who underwent either VLNT or LVA for chronic BCRL between January 1st, 2016, and December 31st, 2023, who had a with a minimum of follow-up period of one year and written informed consent. Patients with other causes of lymphedema, with bilateral treatment, with adjunctive liposuction and/or without written informed consent were excluded from this study.

The primary outcome assessed relative reduction rates over time, serving as a surrogate marker for the effectiveness of the surgical technique. The circumferences of lymphedematous and unaffected arms were measured by a team of specially trained plastic surgery residents at distinct reference points, defined as the maximum hand width, the wrist, and five additional points at 10 cm intervals up to 40–50 cm proximal to the wrist [33, 34]. Measurements were recorded preoperatively and during outpatient follow-up visits at 3, 6, 12, 18 and 24 months postoperatively. In addition to circumferential measurements, preoperative imaging with indocyanine green (ICG) lymphography and/or lymphatic MRI was performed in every patient to confirm the diagnosis of lymphedema and to visualize superficial lymphatic vessels, hence allowing for more appropriate choice of surgical technique. A comprehensive review of patients’ medical chart was conducted to collect data on demographic and clinical parameters, including Body Mass Index (BMI) and postoperative complications. The later were graded following Clavien-Dindo [35].

### Definition of lymphedema

The definition of chronic lymphedema was based on the criteria provided by the international society of lymphology, which specifies an inter-limb volume difference of > 10% or excess volume persisting for more than 3 months [36].

### Average arm circumferences

To visualize and evaluate the absolute changes in arm size, the average arm circumference (AAC) was calculated at each time point using the following formula:

### Relative reduction rates

To accurately assess treatment effectiveness compared to the unaffected arm while accounting for changes in body composition over time, relative reduction rates (RRR) were calculated for each postoperative follow-up using the following formula [37]:

$$RRR = \left( {1 - \frac{{\frac{{\emptyset {\text{Circumference month x }}\left( {\text{affected arm}} \right)}}{{\emptyset {\text{Circumference preop}}.{ }\left( {\text{affected arm}} \right)}}{ }}}{{\frac{{\emptyset {\text{Circumference month x }}\left( {\text{unaffected arm}} \right)}}{{\emptyset {\text{Circumference preop}}.{ }\left( {\text{unaffected arm}} \right)}}}}} \right) \times 100$$

### Relative excess reduction rates

Relative excess reduction rates (RERR) were used to quantify changes in excess volume adjusted to the unaffected limb and were calculated for each postoperative follow-up with the following formula:

$${\text{RERR = }}\left( {1 - \frac{{\emptyset {\text{Circumference month}} x \left( {\text{affected arm}} \right) - \emptyset {\text{Circumference month }}x \left( {\text{unaffected arm}} \right)}}{{\left| {\emptyset {\text{Circumference preop}}. \left( {\text{affected arm}} \right) - \emptyset {\text{Circumference preop}}. \left( {\text{unaffected arm}} \right)} \right|}}} \right) \times 100$$

### Statistical analysis

Statistical analysis was conducted using R software (version 4.2.2). *p*-values ≤ 0.05 were considered statistically significant. Patients’ characteristics were calculated as mean and standard deviations. Comparative analyses of continuous variables between VLNT and LVA groups were performed using linear mixed effects models.

## Results

### Patient characteristics

A total of 112 patients were enrolled in this study, of which 111 (99.1%) were female and one (0.9%) was male (Table 1). All patients developed secondary lymphedema following breast cancer treatment. VLNT surgery was performed in 70 (62.5%) patients, involving inguinal lymph nodes in 66 (94.3%) cases. Abdominal lymph nodes were utilized for the remaining four (5.7%) cases, of which three (4.3%) were omental and one (1.4%) was jejunal mesenteric. The LVA group comprised 42 (37.5%) patients and featured an average of 2.3 ± 0.7 anastomoses per patient, predominantly (97.6%) of the side-to-end type.

**Table 1:** Patients’ characteristics of the VLNT and LVA groups. The values are demonstrated as mean (standard deviation) and number (%).

	All patients (*n* = 112)	VLNT (*n* = 70)	LVA (*n* = 42)
Age	55.0 (9.1)	54.3 (9.1)	56.0 (9.0)
*Sex*			
Female Male	111 (99.1%) 1 (0.9%)	70 (100%) 0 (0.0%)	41 (97.6%) 1 (2.4%)
BMI	26.8 (4.3)	27.3 (4.0)	26.1 (4.8)
Surgery duration (min.)	186.4 (42.1)	211.4 (35.0)	163.2 (34.8)
*Lymph node harvesting site*			
Inguinal Omental Jejunal–mesenteric		66 (94.3%) 3 (4.3%) 1 (1.4%)	
Number of anastomoses			2.3 (0.7)

Age distribution was comparable across both groups, with a mean age of 54.3 ± 9.1 years for VLNT patients and 56.0 ± 9.0 years for those undergoing LVA. BMI values also exhibited similarity across both cohorts, with 27.3 ± 4.0 for VLNT and 26.1 ± 4.8 for LVA. The minimum follow-up period of one year was completed by 107 (95.5%) patients, thus excluding five (4.5%) patients from subsequent circumferential analysis due to incomplete duration of follow-up.

### Absolute reduction rates

**Fig. 1:**
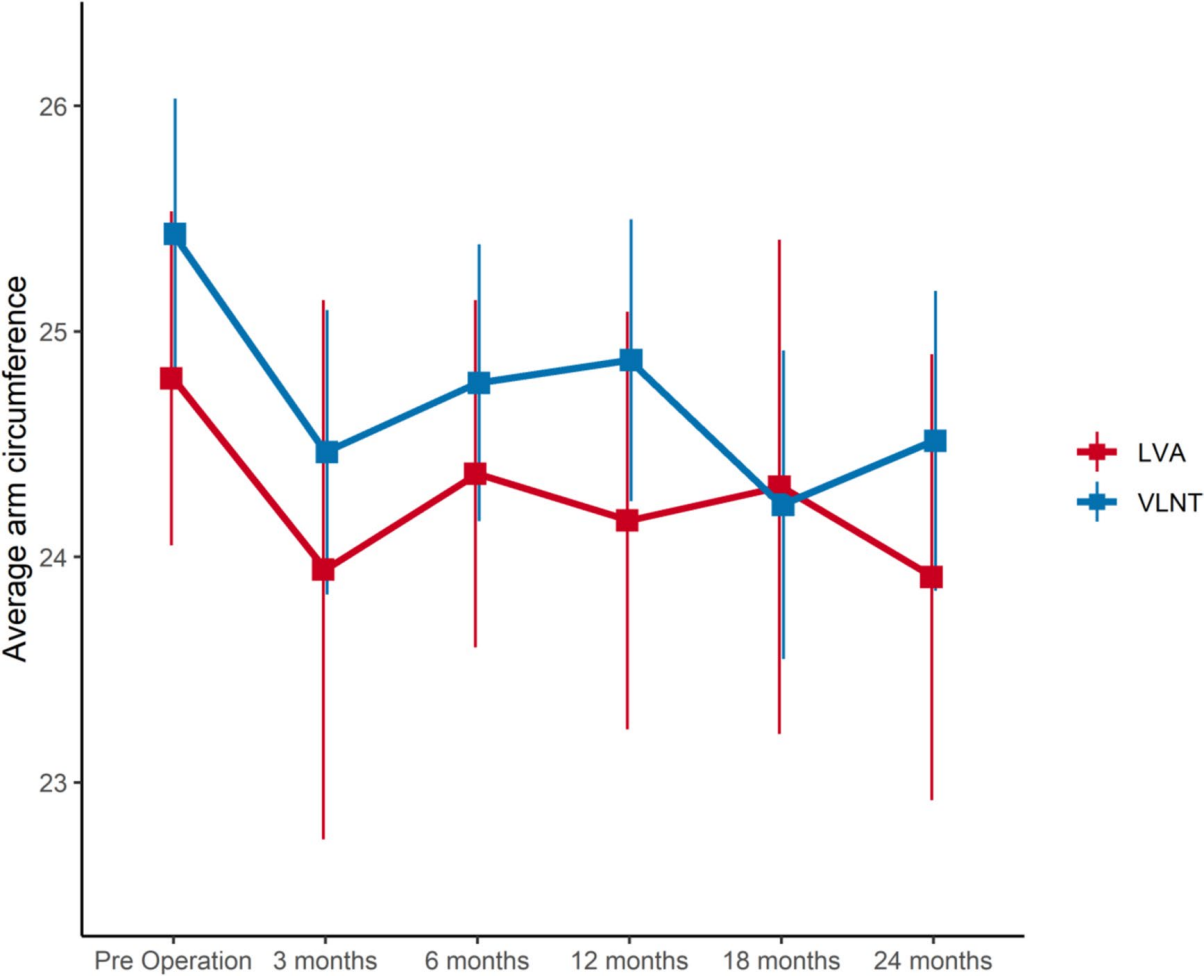
Average arm circumferences [cm] of VLNT and LVA over time

The VLNT group demonstrated slightly larger preoperative mean circumferences in comparison to the LVA group, with preoperative circumferential measurements averaging 25.43 cm for VLNT and 24.79 cm for LVA (Fig. 1, Table 2). Both, VLNT and LVA, significantly reduced arm sizes within three months post-surgery. Specifically, VLNT yielded an average reduction of 0.97 cm, while LVA exhibited an average reduction of 0.85 cm during this early postoperative period. Concluding the 24-month follow-up period, VLNT and LVA achieved absolute reductions of 0.92 cm and 0.88 cm, respectively.

**Table 2:** Average arm circumferences [cm] between the VLNT and LVA groups

	VLNT		LVA	
Time after surgery	Estimate	95% CI	Estimate	95% CI
Preoperative	25.43	24.83–26.03	24.79	24.05–25.53
3 months	24.46	23.83–25.09	23.94	22.75–25.14
6 months	24.77	24.16–25.39	24.37	23.60–25.14
12 months	24.87	24.25–25.50	24.16	23.24–25.09
18 months	24.23	23.54–24.91	24.31	23.21–25.41
24 months	24.51	23.85–25.18	23.91	22.92–24.90

### Relative reduction rates

**Fig. 2:**
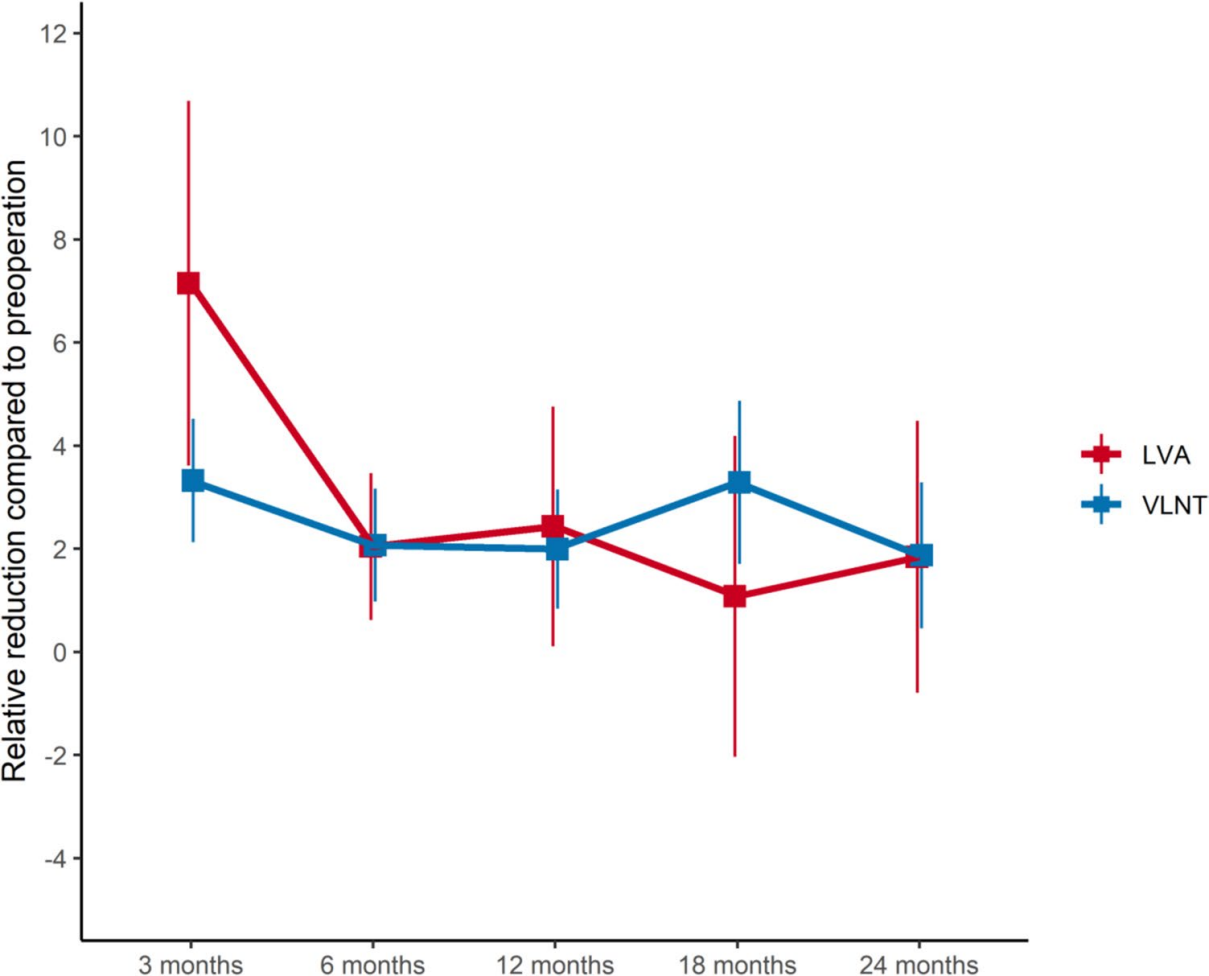
Relative reduction rates [%] for VLNT and LVA over time

LVA achieved its maximum improvement of 7.15% relative reduction in arm circumference three months postoperatively (Fig. 2, Table 3). Notably, its effect significantly decreased to 2.04% at the six-month follow-up. Subsequent fluctuations observed were minor and statistically non-significant, ultimately achieving a final relative reduction rate of 1.84% after 24 months. In comparison, VLNT demonstrated an initial improvement rate of 3.32% at three-month assessment. This rate slightly declined to 2.07% at the six-month mark. Interestingly, VLNT re-gained effectiveness of 3.29% reduction after 18 months. Upon conclusion of the follow-up period, VLNT maintained a final relative reduction rate of 1.88%.

**Table 3:** Relative reduction rates [%] between the VLNT and LVA groups

	VLNT		LVA	
Time after surgery	Estimate	95% CI	Estimate	95% CI
3 months	3.32	2.13–4.52	7.15	3.61–10.69
6 months	2.07	0.97–3.16	2.04	0.62–3.47
12 months	2.00	0.84–3.15	2.44	0.11–4.77
18 months	3.29	1.70–4.87	1.08	− 2.03–4.19
24 months	1.88	0.47– 3.30	1.84	− 0.79–4.48

### Relative excess reduction rates

**Fig. 3:**
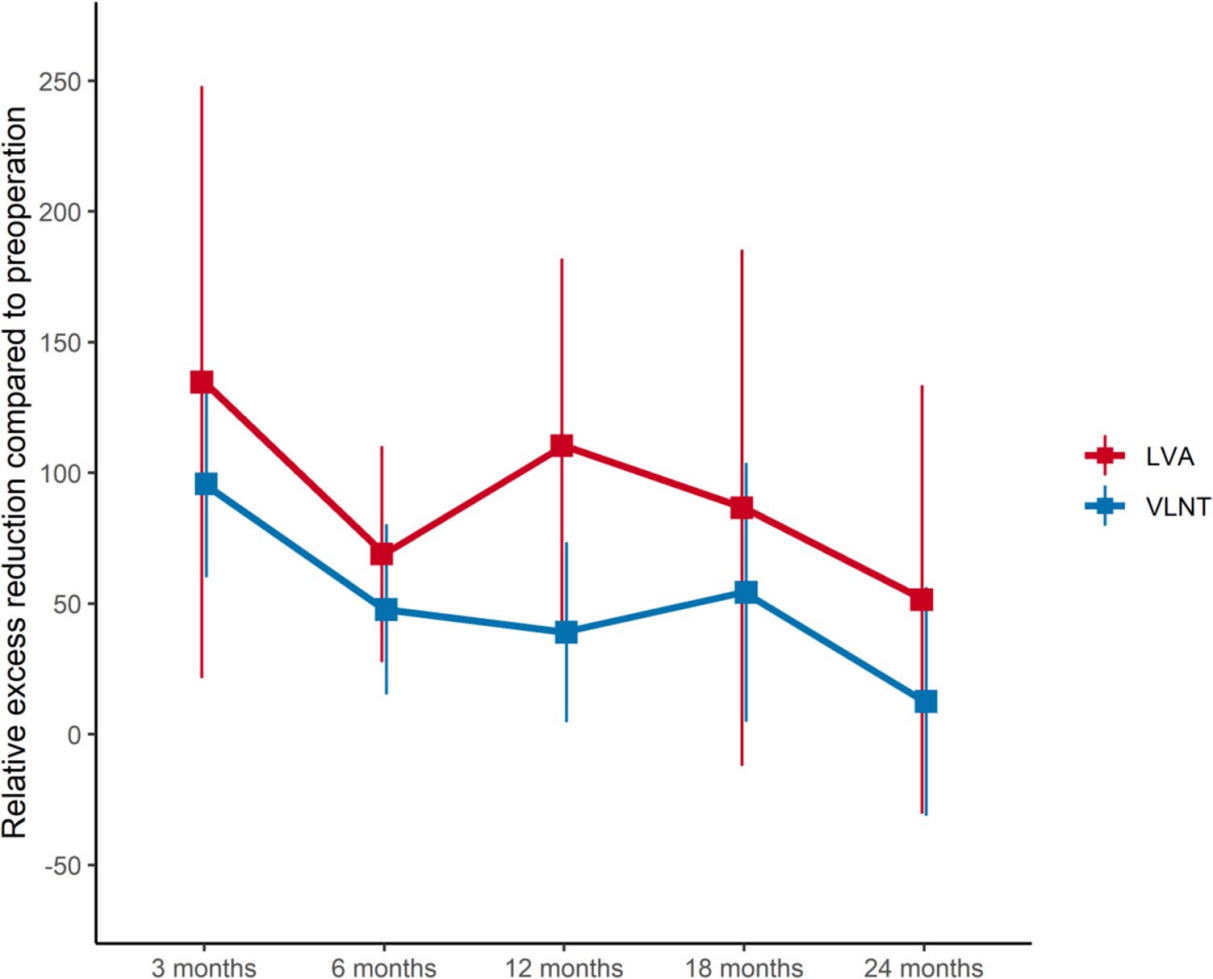
Relative excess reduction rates [%] for VLNT and LVA over time

Both VLNT and LVA achieved significant reductions in excess arm volume (Fig. 3). Notably, there was considerable interindividual variability in RERR, as indicated by large 95% confidence intervals (Table 4). After three months, both techniques achieved a near-total removal of the surplus volume, with certain patients even demonstrating slimmer arms than contralaterally. However, a re-gain of arm volume was observed after six months, mirroring the decrease in effectiveness observed within the relative reduction rates. By the end of the two-year follow-up, the potency of both interventions further declined, with mean RERRs of 51.2% (95% CI -30.79—133.29) for LVA and 12.52 (95% CI -31.20—56.25) for VLNT.

**Table 4:** Relative excess reduction rates [%] between the VLNT and LVA groups

	VLNT		LVA	
Time after surgery	Estimate	95% CI	Estimate	95% CI
3 months	95.76	60.01–131.51	134.51	21.20–247.82
6 months	47.59	14.99–80.19	68.92	27.59–110.26
12 months	39.01	4.57–73.45	110.45	38.77–182.13
18 months	54.01	4.51–103.52	87.60	− 11.21–186.41
24 months	12.52	− 31.21–56.27	51.25	− 30.79–133.29

### Manual lymphatic drainage

Preoperatively, patients scheduled for VLNT and LVA required an average of 2.1 ± 0.7 and 1.6 ± 1.1 manual lymphatic drainages (MLD) per week, respectively (*p* = 0.11). Six months postoperatively, both groups experienced a reduction in MLD frequency, averaging 1.8 ± 0.8 MLDs per week for VLNT and 1.5 ± 0.8 for LVA (*p* = 0.16). By the one-year follow-up, the MLD frequency stabilized for VLNT patients at 2.0 ± 0.6 MLDs per week, whereas LVA patients showed further reductions to 1.3 ± 0.6 per week (*p* = 0.17) Although data at the two-year follow-up was inconsistently reported, the average number of MLDs per week appeared similar between groups, with VLNT patients at 2.0 ± 0.7 and LVA patients at 1.5 ± 0.7 (*p* = 0.49).

### Complications

A total of ten (14.3%) complications arose from the VLNT group, encompassing five (7.2%) seromas, two hematomas (2.9%), one fat necrosis (1.4%), one partial loss (1.4%), and one total flap loss (1.4%). The majority (70%) of complications classified as Clavien-Dindo grade II or lower and, hence, were mild only. In comparison, no complications were observed in the LVA group.

## Discussion

Vascularized lymph node transfer (VLNT) is thought to enhance lymphatic drainage through lymphangiogenesis, facilitating the formation of novel collateral pathways [29, 30]. This process, which is generally viewed as time-consuming, finds support in experimental indocyanine green (ICG) studies demonstrating pumping of lymphatic fluid from the interstitium through the transplanted lymph node to the pedicle vein in the postoperative follow-ups [31, 32]. Despite this, rapid improvements in certain patients suggest an alternative, more immediate mechanism of action, potentially explained by a release of scarring in previously damaged soft tissue [38]. These rapid improvements, however, remain underreported, thus leaving the precise pathophysiological mechanisms of VLNT yet to be fully explored.

In this comparative study of vascularized lymph node transfer and lymphaticovenous anastomosis (LVA), both groups achieved notable reductions in average arm circumferences within the first three months (Fig. 1, Table 2). Subsequent follow-up revealed minor fluctuations in arm size for both groups, underscoring the long-term potential of both treatments.

Relative reduction rates (RRRs) compare volumetric changes of a lymphedematous extremity to changes in the contralateral limb over a defined period of time, hence correcting for any changes in BMI. In contrast, relative excess reduction rates (RERR) accurately quantify reductions in surplus arm volume. During the initial three months, LVA outperformed VLNT, with RRRs of 7.15% vs. 3.32% and RERRs of 134.51% vs. 95.76%, respectively. This initial disparity underscores the rapid onset of therapeutic effects associated with LVA, aligning with current literature (Fig. 2, Table 3) [13, 14]. However, LVA’s initial effectiveness declined to a RRR of 2.04% after six months, mirroring results reported by Qiu et al. [13]. Simultaneously, a re-gain in excess limb volume was observed (Fig. 3, Table 4). This pattern could potentially be linked to a reduction in the number of patent lymphaticovenous anastomoses within the first year, as suggested by prior studies [15, 23]. The subsequent stabilization of arm circumferences post-six months suggests that anastomotic closure primarily occurs during the early postoperative phase. Consequently, vessels that remain patent after this critical period are more likely to preserve their long-term patency, thereby allowing for LVA’s long-term positive effects. These observations align with existing literature, which correlates the numbers of lymphaticovenous anastomoses per limb with the long-term effectiveness of LVA [39, 40]. In this study however, no clear correlation between the number of surgical anastomoses and the relative reduction rate over varying time intervals could be observed, likely attributable to the marked predominance of two anastomoses per patient in our cohort. While promising, these preliminary conclusions require future validation, ideally through comprehensive indocyanine green (ICG) lymphography studies with consistent follow-up in the early postoperative period [41].

Interestingly, VLNT already exhibited a notable RRR of 3.32% and RERR of 95.7% at the three-month follow-up. As all patients with simultaneous liposuction were excluded from this study, these observations challenge the current understanding of VLNT’s purely delayed mechanism of action suggested in the majority of the existing literature [29–31]. These findings align with similar papers, which demonstrate unexpected volume reductions within the first month after VLNT [33, 42]. However, these observations could potentially be influenced by a more coherent wearing of compression garments in the early postoperative period [43, 44]. Following minor and non-significant fluctuations throughout the follow-up period, VLNT demonstrated a surge in effectiveness with a relative reduction rate of 3.29% with a concurrent reduction in excess arm volume at 18 months postoperatively, which is consistent with the hypothesized mechanism of slow lymphangiogenesis via secretion of vascular endothelial growth factors [30, 31]. VLNT and LVA were similarly effective after 24 months, demonstrated by RRRs of 1.88% and 1.84%, respectively. The minimal difference in effectiveness suggests that VLNT and LVA are maximally effective when correctly applied in a stage-specific manner [19, 45, 46]. Additionally, the comprehension of VLNT’s and LVA’s temporal dynamics is crucial for managing patient expectations. Notably, the relative reduction rates observed throughout the follow-up period could be even underestimated, due to the effect of patients wearing compression garments before surgery compared to the discontinuation or reduction of wearing the garments after successful improvement following lymphatic surgery.

Furthermore, substantial decreases in manual lymphatic drainage (MLD) frequency were observed over time. Preoperatively, patients undergoing VLNT required slightly more MLD sessions per week compared to the LVA group (2.1 ± 0.7 vs. 1.6 ± 1.1, *p* = 0.11). However, six months postoperatively, MLD frequency decreased for both groups, averaging 1.8 ± 0.8 per week for VLNT and 1.5 ± 0.8 for LVA (*p* = 0.16), indicating early improvements in lymphatic function. By one year, MLD frequency stabilized in VLNT patients at 2.0 ± 0.6 MLDs per week, while LVA patients experienced further reductions to 1.3 ± 0.6 per week (*p* = 0.17). Although the data were less consistent at the two-year follow-up, MLD frequency remained comparable between groups, with VLNT at 2.0 ± 0.7 and LVA at 1.5 ± 0.7 (*p* = 0.49). These findings align with previous studies demonstrating reduced MLD frequency after lymphatic reconstruction, supporting these interventions as effective treatment options that lessen dependency on MLD and thereby reduce the associated financial burdens associated with lymphedema [13, 47–49]. Consequently, the observed improvements following VLNT and LVA may be even more significant when accounting for the decreased need for MLD.

In summary, the initial three-month reduction observed with VLNT was an unforeseen yet significant finding, suggesting an earlier onset of therapeutic impact than traditionally understood. Its re-gain in effectiveness at 18 months post-treatment supports the currently understood delayed but durable response, in contrast to the more immediate but partially transient effect of LVA. Hence, we suggest that the future decision-making process and patient counseling should include the temporal profile of both surgical techniques, ensuring that the chosen intervention aligns with the patient's individual needs.

### Study strengths and limitation

This study reveals significant insights into temporal effectiveness profiles of VLNT and LVA in lymphedema management and as such contributes to more personalized treatment strategies for chronic BCRL. The main strength of this study is its relatively large sample size of 112 patients, increasing the generalizability of these novel results. However, follow-up imaging to assess for correlation between clinical improvements and changes in classic ICG patterns was not routinely performed, which would have provided additional insights. Moreover, the specific individual performing the circumferential measurements was not recorded in the database, limiting the ability to assess inter- and intra-rater reliability. Finally, this study solely focused on quantitative data, overlooking patient-reported outcomes that are crucial for proper assessment of patient satisfaction and treatment effectiveness. While this study offers important novel insights into lymphedema treatment, it also underscores the need for future randomized, controlled, multicenter studies including patient-reported outcome measures (PROMs).

## Conclusions

VLNT and LVA both improve patient outcomes, yet their temporal effectiveness profiles differ significantly. While LVA demonstrates rapid initial improvement, its long-term potency appears to wane, possibly due to early closure of lymphaticovenous anastomoses. Notably, VLNT also demonstrates early effectiveness and sustains a slightly higher long-term effectiveness compared to LVA.

## Data Availability

The data from our encrypted databank that was analyzed as part of this study is not publicly available pursuant to the ethics committee approval by the “Ethikkommission Nordwest und Zentralschweiz EKNZ” in Switzerland due to their sensitive nature. Nevertheless, the data can be obtained from the corresponding author upon reasonable request.
